# Prognostic value of chemotherapy-induced leukopenia in small-cell lung cancer

**DOI:** 10.7497/j.issn.2095-3941.2013.02.005

**Published:** 2013-06

**Authors:** Wei Liu, Cui-Cui Zhang, Kai Li

**Affiliations:** Department of Thoracic Oncology, Key Laboratory of Cancer Prevention and Treatment of Tianjin City, Tianjin Lung Cancer Center, Tianjin Medical University Cancer Institute and Hospital, Tianjin 300060, China

**Keywords:** Small-cell lung cancer (SCLC), leukopenia, prognosis

## Abstract

**Objective:**

Chemotherapy is the standard treatment for small-cell lung cancer (SCLC), and leukopenia is a common side effect. This study assesses whether chemotherapy-induced leukopenia is a predictor of efficacy and whether it is associated with the survival of SCLC patients.

**Methods:**

A retrospective analysis was conducted on data from 445 patients with SCLC who received standard chemotherapy for 4 to 10 cycles. The World Health Organization grading system classifies leukopenia during chemotherapy as follows: absent (grade 0), mild (grades 1 and 2), or severe (grades 3 and 4). The primary endpoint is overall survival (OS).

**Results:**

The association between chemotherapy-induced leukopenia and OS was assessed. According to a multivariate Cox model with time-varying covariates, the hazard ratio of death was significantly lower among patients with mild leukopenia than among patients with severe leukopenia at 0.687 (0.506 to 0.943) and 1.414 (1.147 to 1.744), respectively. The median survival was 13 months (95% CI: 11 to 15 months) for patients who did not experience leukopenia, 17 months (95% CI: 14 to 18 months) for those with mild leukopenia, and 14 months (95% CI: 13 to 16 months) for those with severe leukopenia (absent *vs*. mild *vs*. severe leukopenia, *P*=0.047).

**Conclusion:**

Leukopenia during chemotherapy is associated with the survival of SCLC patients. Mild leukopenia is strongly associated with longer survival time.

## Introduction

Lung cancer continues to be a severe global health problem and the leading cause of cancer deaths worldwide. Small-cell lung cancer (SCLC) accounts for approximately 15% to 20% of all bronchogenic carcinomas. The main treatment for SCLC involves chemotherapeutic drugs that have various side effects, of which leukopenia is the most common. Leukopenia during cytotoxic chemotherapy is positively associated with survival in several types of cancer^1^^-^^3^, suggesting that the hematologic toxicity caused by drugs is a biological measure of drug activity and a predictor of treatment efficiency. In this study, data from 445 SCLC patients were reviewed to assess whether chemotherapy-induced leukopenia is associated with survival.

## Patients and methods

### Clinical data

All patients diagnosed with SCLC, who registered at Tianjin Medical University Cancer Institute and Hospital (TMUCIH) between February 2005 and December 2010, were searched from the TMUCIH database. This study identified 445 patients who received 4 to 10 cycles of chemotherapy with EP and who met the following inclusion criteria: histologically/cytologically confirmed to have SCLC; aged 18 to 80 years; ECOG performance status of 0, 1, or 2; adequate bone marrow function (leukocytes >4,000/µL, neutrophil >2,000/µL, platelet >100×10^9^/L, hemoglobin >9.0 g/dL); without serious cardiovascular disease; and with no previous or other concomitant malignant disease. Treatment was discontinued if the patient developed unacceptable toxic effects due to refusal of treatment. All patients received standard chemotherapy of EP regimen (etoposide 100 mg/m^2^ and cisplatin 75 mg/m^2^, with every cycle of 21 days). Limited-stage disease (LD) patients with good performance status received thoracic radiotherapy whereas LD patients in complete remission received prophylactic cranial irradiation (PCI) during their evaluation four weeks after chemotherapy. If at least one criterion is not met on day 1 of each chemotherapeutic cycle, chemotherapy is postponed for up to a maximum of two weeks. By protocol, dose reductions were not planned. This study was approved by the Ethics Committee of TMUCIH. Written informed consent was obtained from all patients.

### Evaluation of leukopenia, supportive therapy, and other organ toxicities

Routine blood counts were conducted every chemotherapy cycle, at day 1 before treatment and around day 7 and 14. Leukopenia and other organ toxicities, such as liver and renal toxicities, were graded in accordance with the World Health Organization criteria (version 3.2). The worst status of leukopenia was recorded in each treatment cycle. Blood routine examination was conducted daily. Granulocyte-colony-stimulating factor (G-CSF) was used in patients with grade 4 leukopenia until their leukocyte count reached >4.0×10^9^/L or any significant level of persistent non-hematologic toxicity appeared. The patients were grouped into three according to degree of leukopenia: absent (grade 0), mild (grade 1-2), and severe (grade 3-4).

### Statistical analysis

Overall survival (OS) was the primary endpoint of the analysis. Outcome intervals were calculated from the date of therapy initiation to the date of death or last follow-up for patients who were alive without progression. Kaplan-Meier plots were used to estimate OS. The effect of single variables on OS was assessed using log-rank test and Kaplan-Meier plots. A Chi-square test assessed the association between the categorical variables and the degree of leukopenia. The hazard ratios (HR) of death and 95% CI were estimated using Cox multivariable regression analysis. A backward likelihood-ratio test approach was adopted in building the Cox survival model; gender, stage or extent of the disease (limited-stage *vs.* extensive-stage), performance status (2 *vs.* 0 to 1), thoracic radiotherapy history, PCI history, and degree of leukopenia (mild *vs.* none, severe *vs.* none, mild *vs.* severe) were the final covariates in the Cox analysis. All statistical tests were two-tailed, and *P* values of less than 0.05 were considered significant. Analyses were performed using SPSS 19.0.

## Results

### Patients, treatments and toxicities

Our retrospective analysis included 445 SCLC patients who received 4 to 10 cycles of chemotherapy in TMUCIH during February 2005 and December 2010 and who fulfilled the eligibility criteria. Among these patients, 340 (76%) were males and 105 (24%) were females. Their ages ranged from 18 to 80, with a median age of 59 years. The ECOG PS of most patients was from 0 to 1. According to extent of the disease, 233 (52%) were classified as LD patients whereas 212 (48%) were classified as ED patients. Seventy-seven patients had mixed stages of SCLC (**Table 1**).

**Table 1:** Baseline demographics and clinical characteristics in patients and in subgroups based on the grade of leukopenia

Characteristics	All patients *n*=445	Leukopenia (grade 0-4)					*P*
		0	1	2	3	4	
Age, years	59 (18-80)	57 (30-78)	60 (18-78)	58 (35-80)	59 (28-81)	62 (40-79)	0.063
Gender							0.042
Male	340	62	53	80	86	59	
Female	105	10	14	35	24	22	
Performance Score							0.04
0	345	61	52	97	86	59	
1	81	9	14	16	18	14	
2	19	2	1	2	6	8	
Stage							0.022
Limited	233	48	35	62	56	32	
Extensive	212	24	32	53	54	49	
Pathology							0.094
Small-cell	368	55	56	100	85	72	
Mixed small-cell	77	17	11	15	25	9	
Tumor location							0.739
Central	247	38	36	68	57	48	
Peripheral	198	34	31	47	53	33	
Radiotherapy History							0.189
Yes	220	32	34	66	55	33	
No	225	40	33	49	55	48	
Chemotherapy cycles							0.001
<6	195	49	35	46	34	31	
≥6	250	23	32	69	76	50	

**Figure 1:**
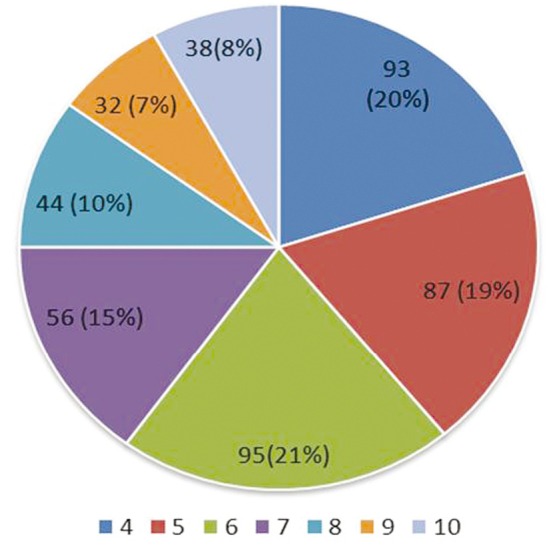
Number of chemotherapeutic cycles recorded in 445 patients.

The number of chemotherapeutic cycles recorded in the 445 patients is shown in **Figure 1**, with the mean number of cycles as 6. Thoracic radiotherapy was administered to 220 patients; the median dose of radiotherapy was 46 Gy. Most patients received either 40 Gy in 15 fractions (35%) or 50 Gy in 25 fractions (43%). Forty-five patients received prophylactic cranial irradiation (30 Gy in 10 fractions). Mild leukopenia (grade 1-2) was noted in 182 (41%) of the 445 patients, whereas severe leukopenia (grade 3-4) was noted in 191 (43%) patients. The other 72 (16%) patients did not experience leukopenia. Eighty-one patients with grade 4 leukopenia received G-CSF. No other cases of organ toxicity greater than grade 2 (excluding alopecia) were recorded during the treatment.

### Relationship between leukopenia and prognosis

**Figure 2:**
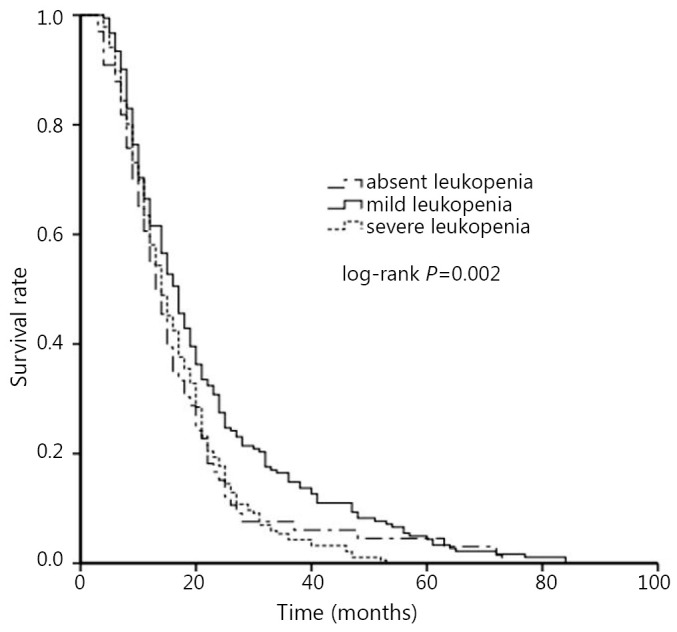
Kaplan-Meier survival curves of the three groups of leucopenia.

The number of deaths recorded was 378, of which 369 (97%) were associated with tumor. Among the 85 patients that died from serious infection, 62 had severe leukopenia and 23 had mild leukopenia. Nine cases of death were traced to other causes. The median OS for all patients was 15 months. The univariate analysis using Kaplan-Meier method and the comparison using log-rank test showed that the degree of leukopenia in patients was significantly associated with OS (*P*=0.009, **Figure 2**). The median OS was 17 months (95% CI: 14 to 18 months) for cases of mild leukopenia, 13 months (95% CI: 11 to 15 months) for cases where leukopenia was absent, and 14 months for cases of severe leukopenia (95% CI: 13 to 16) (mild *vs.* absent *vs.* severe, *P*=0.047; mild *vs.* absent, *P*=0.039; mild *vs.* severe, *P*=0.004; absent *vs.* severe, *P*=0.435). Univariate analysis revealed other prognostic indicators: smoking history (*P*=0.022), performance status (*P*<0.001), extent of disease (*P*<0.001), thoracic radiotherapy history (*P*<0.001), and PCI history (*P*<0.001).

The results of the Cox regression analysis for the multivariate analysis are presented in **Table 2**. Leukopenia was identified as one of five independent prognosis factors (*P*=0.002). The HR for groups where leukopenia was mild (grade 1-2) and absent (grade 0) was 0.687 (95% CI: 0.506 to 0.943, *P*=0.017) whereas the HR for groups with severe leukopenia (grade 3-4) and mild leukopenia (grade 1-2) was 1.414 (95% CI: 1.147 to 1.744; *P*=0.001). However, the HR for groups where leukopenia was severe (grade 3-4) and absent was 0.901 (95% CI: 0.669 to 1.214; *P*=0.494). A significant difference (*P*=0.013) was noted in the HR of groups where leukopenia was mild versus absent and that of groups where leukopenia was severe versus absent. No significant difference (*P*=0.988) was found in the HR of groups where leukopenia was absent *vs.* severe.

**Table 2:** Multivariate Cox model for the association between survival and chemotherapy-induced leukopenia

Characteristics	Hazard ratio (95% CI)	*P*
Leukopenia		0.002
Mild *vs*. absence	0.687 (0.506-0.943)	0.017
Severe *vs*. mild	1.414 (1.147-1.744)	0.001
Severe *vs*. absence	0.901 (0.669-1.214)	0.494
Gender		
Male *vs*. female	1.335 (1.042-1.709)	0.022
PS status		
2 *vs*. 0-1	1.591 (1.274-1.988)	*<*0.001
Stage		
Extensive *vs*. limited	1.959 (1.589-2.416)	*<*0.001
Thoracic radiotherapy		
Yes *vs*. no	0.806 (0.649-0.996)	0.046
PCI history		
Yes *vs*. no	0.591 (0.379-0.920)	0.020

Treatment was discontinued in 67 (15%) of the 445 patients in the study because of intolerable toxic effects (mean cycle was 4.5; median OS was 13 months). Leukopenia was absent in 9 patients (13%); patients with mild and severe leukopenia totaled 16 (25%) and 42 (62%), respectively. However, among the 378 patients who received regular treatment, 40 (16%) were reported to have an absence of leukopenia, whereas 166 (44%) and 152 (40%) patients were reported to have mild and severe leukopenia, respectively. The extent of leukopenia in the 67 patients whose treatment was discontinued differed from those who received regular chemotherapies (*P*=0.006). Furthermore, the incidence of severe leukopenia in these 67 patients was higher than in the other 378 patients (*P*=0.001); however, no significant difference was found in the incidence of patients with an absence of and mild leukopenia, at *P*=0.283 and 0.094, respectively. Longer OS was reported in cases of mild leukopenia during treatment, but no change in OS was reported after the exclusion of the 67 patients, whose treatment was discontinued, from the total number of patients in the study (*P*=0.008 for leukopenia in the Cox model).

Aside from leukopenia, other independent predictive factors for OS are gender, performance status, extent of disease, history of thoracic radiotherapy, and PCI (**Table 2**). A synergistic effect of chemotherapy cycles is possible in leukopenia; however, the mixed variable multiplied by the two variables was ruled out by the Cox regression analysis equation, suggesting no synergistic effect on the two variables.

### Relationship between leukopenia and other clinical parameters

Chi-square test results (**Table 1**) showed that the occurrence of leukopenia was more likely in patients who were female (*P*=0.042), as well as in those with ECOG PS ≥2 (*P*=0.04), in later stages of the disease (*P*=0.022), and with increased cycles of chemotherapy (*P*=0.001). Similar analysis was performed to determine hematologic toxicity; hemoglobin decrease, thrombocytopenia, and neutropenia. However, the data on these variables were no longer shown as they were of less significance to the analysis than leukopenia.

## Discussion

Mild chemotherapy-induced leukopenia (grade 1-2) is an independent prognostic factor for increased survival, whereas severe leukopenia represents higher risk of death. Of the 191 patients with severe leukopenia, 143 (75%) had severe neutropenia; among the latter, 62 (32%) directly or indirectly died from serious infection. Only 23 (9%) patients died from infection in cases where leukopenia was either absent or mild. The severity of leukopenia increased the risk of serious infection, as shown in the unfavorable prognosis of patients with SCLC.

The idea that neutropenia may be a surrogate indicator of the biological activity of drugs has been supported in several reports^4^. Since the late 1990s, a series of reports have suggested better outcomes of both post-operative and neoadjuvant chemotherapies of breast cancer in patients with myelosuppression^5^^-^^9^. Chemotherapy-induced neutropenia during treatment is associated with higher probability of treatment response and better survival in colon and gastric cancer patients^2^^,^^3^^,^^10^. A retrospective analysis^11^ of four clinical trials of SCLC concluded that women have higher incidence of toxic effects, increased response, and longer survival than men. Di Maio *et al.*^12^ concluded that both mild (grade 1-2) and severe (grade 3-4) neutropenia in advanced non-small-cell lung cancer (NCLC) may be surrogate markers of adequate chemotherapy dosage; the lack of neutropenia indicates underdosage whereas severe neutropenia indicates overdosage.

Hematology toxicity is one of the most important reasons for limiting dosage. In cases where leukopenia is mild, rather than absent or severe, during chemotherapies, patients showed favorable survival, indicating that an optimal dosage of drugs leads to better survival. A possible cause of under- or overdosage of drugs is the induction of body surface area for determining the dose. The optimal dose defined by the body surface area may be insufficient in some cases. Poor correlations were observed between the body surface area and the pharmacokinetic variables of most cytotoxic drugstores because of the variability in patients’ metabolisms^13^^-^^16^. The pharmacokinetics of vinorelbine was associated not only with body surface area, but also with body weight, serum creatinine, platelet count, and sex^17^. Similarly, the pharmacokinetics of gemcitabine was strongly affected by sex^18^. Simron *et al.*^11^ reported that females experience significantly more obvious hematologic toxicity than males. Moreover, among same-age patients and those with normal liver biochemistry, women have a lower rate of clearance for doxorubicin and gemcitabine^19^^,^^20^. Another possible reason for the higher incidence of hematologic toxicity in women is their higher baseline body mass index (BMI) than that of men because of increased body fat, which may affect the distribution of cytotoxic drug and increase potential toxicity^21^. Obese patients with high BMI also showed a lower drug clearance and a resultant increase in the elimination half-life of doxorubicin and cyclophosphamide^22^^,^^23^. Furthermore, Gurney reported that, aside from BMI, epirubicin PK indices or neutropenia were not correlated with serum aminotransferase levels or other biochemical liver function indices, creatinine, and other clinical factors^24^. However, activated partial thromboplastin time was associated with serum transferrin. In our study, other organ toxicities under grade 2 do not have a crucial impact on the PK of drugs.

As no prophylactic application was stipulated in protocols, G-CSF was prescribed only in patients with grade 4 leukopenia; such G-CSF treatment could be one of the reasons for the increased death risk in patients with severe leukopenia. Therefore, supplementing chemotherapy with G-CSF could stimulate angiogenesis and promote tumor growth because of the potential contribution of bone marrow-derived endothelial progenitor cells^25^^,^^26^.

Chemotherapy-induced myelosuppression is the common reason for reduced dosage and discontinued treatment which, in turn, lead to patients’ lower chance of survival^27^^-^^29^. In our study, 67 patients whose treatment were discontinued or yielded unfavorable outcomes showed a median OS of only 13 months, which was obviously lower than the OS of 15 months for the 445 patients (*P*=0.0035). However, the low incidence of discontinued treatment (only 15%) in this study suggests that the unchanged result after the exclusion of these 67 patients from the total number of patients can be attributed to the small sample size. Di maio *et al.*^10^ showed that NSCLC patients with severe neutropenia had slightly better chance of survival than those without neutropenia. High-dosage treatment may result in severe toxic effects, with little benefit or poor prognosis in most solid tumors, especially for NSCLC, which has low sensitivity to most cytotoxic drugs. This finding suggests that the right dosage is one that is optimal and that is neither low nor high. Given the complexities of the pharmacokinetics of drugs in patients, the exact dosage for each patient is difficult to calculate. The results of our study suggest that dosage should be tailored based on toxic effect^30^. The optimal drug dosage for SCLC patients is one that induces mild leukopenia during the treatment.

However, our study has several limitations. Adopting retrospective analysis means recording the date of blood counts from the patients’ medical records. Patients with an absence of leukopenia may have experienced occult leukopenia when no blood count data were available. Furthermore, the exact instance of progress free survival (PFS) is difficult to determine in retrospective studies of SCLC; the association of PFS with chemotherapy-induced leukopenia should be assessed. A large randomized trial is needed to demonstrate such associations. Analysis of the correlation of dosage versus toxic effects with plasma concentrations and treatment efficacy could confirm the results of our study.

In conclusion, leukopenia during chemotherapies is associated with the survival of patients with SCLC. Mild, rather than absence of or severe leukopenia, is strongly associated with better prognosis. Therefore, we propose that chemotherapy-induced leukopenia is a surrogate marker for determining the optimal dosage to improve the efficacy of chemotherapy in SCLC. Tailoring dosage based on toxic effect to induce a mild myelosuppression in patients with SCLC leads to better overall survival.
